# Comparison of Different Upfront Two-Stent Strategies for True Coronary Bifurcation Lesions: In-Hospital and 6-Month MACE Results

**DOI:** 10.3390/jcm15166308

**Published:** 2026-08-14

**Authors:** Kerim Esenboga, Alparslan Kurtul, Emre Ozerdem, Yakup Yunus Yamanturk, Halil Gulyigit, Muge Akbulut, Eralp Tutar

**Affiliations:** 1 Department of Cardiology, Ankara University School of Medicine, Ankara 06230, Türkiye; kerimesenboga@yahoo.com (K.E.); yyyamanturk@gmail.com (Y.Y.Y.);; 2 Department of Cardiology, Mustafa Kemal University School of Medicine, Hatay 31060, Türkiye; alparslan.kurtul@mku.edu.tr

**Keywords:** true bifurcation lesion, upfront two-stent strategy, major adverse cardiac events

## Abstract

**Highlights:**

**What are the main findings?**

- No statistically significant differences in MACE were observed among DK-crush, mini-culotte, and T/TAP.
- Previous myocardial infarction was the only independent predictor of MACE (adjusted OR 4.33, 95% CI 1.10–23.61; *p* = 0.035).

**What are the implications of the main findings?**

- Contemporary upfront two-stent techniques may provide comparable short-term clinical outcomes when selected according to lesion characteristics and operator expertise.
- Larger prospective studies with longer follow-up are warranted to define the optimal upfront two-stent strategy for true coronary bifurcation lesions.

**Abstract:**

**Background:** Percutaneous coronary intervention (PCI) remains controversial for coronary bifurcation lesions (CBLs), and over the years, several stent techniques have been developed. Current guidelines on myocardial revascularization recommend an upfront two-stent strategy (U2SS) for complex CBLs. This study was aimed at comparing different upfront two-stenting techniques for true complex CBLs. **Methods:** We retrospectively analyzed 142 consecutive patients with true coronary bifurcation lesions who underwent percutaneous coronary intervention using an upfront two-stent strategy (DK-crush, mini-culotte, or T/TAP) between June 2019 and September 2022. Clinical, angiographic, and procedural characteristics, as well as 6-month clinical outcomes, were compared among the three groups. The primary endpoint was major adverse cardiovascular events (MACE), and independent predictors of MACE were evaluated using a multivariable Firth penalized logistic regression analysis. **Results:** Major adverse cardiac events (MACE) [a composite of death, stent thrombosis, myocardial infarction, and target vessel revascularization] occurred in 6.3% of the study population at a median follow-up period of 6 months. There were no significant differences in the primary clinical outcomes among the groups. Although the DK-crush group had the lowest observed incidence of MACE, this difference did not reach statistical significance (*p* = 0.137). A multivariable Firth penalized logistic regression analysis identified previous myocardial infarction as the only independent predictor of MACE (adjusted OR: 4.33; 95% CI: 1.10–23.61; *p* = 0.035). **Conclusions:** Within the limitations of this study, all upfront two-stent strategies appeared feasible and were associated with relatively low short-term MACE rates. No significant differences in clinical outcomes were observed among the evaluated techniques. Previous myocardial infarction was identified as an independent predictor of MACE in the multivariable analysis.

## 1. Introduction

Coronary bifurcation lesions (CBLs) are defined as coronary artery stenotic lesions involving the origin of significant side branches [1]. Among patients who undergo percutaneous coronary intervention (PCI), the prevalence of CBLs roughly varies from 15% to 20%, which are commonly encountered by cardiologists in daily practice [2]. The optimal PCI strategy for the treatment of CBLs is still controversial, mainly because of the relatively high procedural complications, restenosis rate, and higher rates of long term major adverse cardiac events (MACE) [cardiac death, stent thrombosis, target vessel revascularization, and myocardial infarction] as compared with non-CBLs stenting [3]. In recent times, the provisional stenting strategy has been strongly recommended as the preferred strategy for most CBLs [4,5]. Nevertheless, several randomized controlled trials have indicated that in selected cases, two-stent techniques yield better clinical outcomes, and a systematic upfront two-stent strategy (U2SS) [double kissing (DK) crush/mini-culotte/T or T and small protrusion (TAP)] should be considered in patients with complex CBLs stratified by the DEFINITION criteria [6,7]. Current guidelines on myocardial revascularization recommend an upfront two-stent approach for CBLs with: a large caliber SB (diameter of ≥2.5 mm), and SB lesion lengths greater than 5 mm, or anticipated difficulty in accessing the SB after main vessel (MV) stenting, and a distal true left main bifurcation [8,9]. However, the optimal stenting strategy for true CBLs is still a matter of debate because little is known about the comparison of outcomes of the different U2SSs for true CBLs.

The purpose of this study was, therefore, to compare the in-hospital and 6-month clinical outcomes of different U2SS (stenting of the MV and SB) for the treatment of true CBLs with drug-eluting stents (DES). We also aimed to determine independent predictors of 6-month MACE development after intervention.

## 2. Methods

### 2.1. Study Population

**Figure 1:**
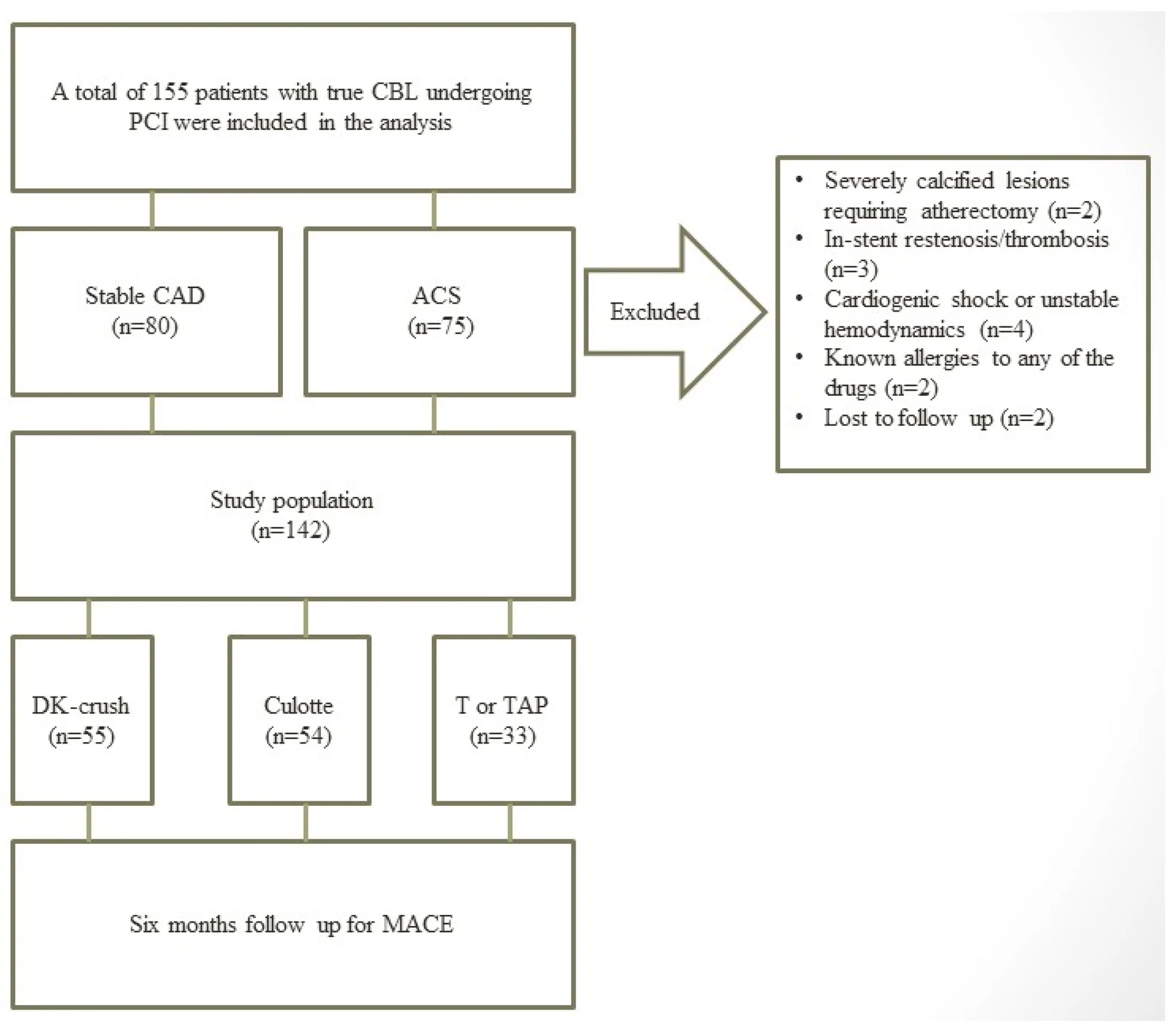
Research flow-chart diagram. Abbreviations: ACS, acute coronary syndrome; CAD, coronary artery disease; CBL, coronary bifurcation lesion; DK, double kissing; MACE, major adverse cardiovascular events; PCI, percutaneous coronary intervention; TAP, T and protrusion.

A total of 155 patients were enrolled retrospectively from June 2019 to September 2022. Patients that exhibited stable coronary artery disease (CAD) or acute coronary syndromes (ACS) and underwent PCI for de novo coronary lesions (diameter stenosis of >70%) at the true bifurcation, with an SB diameter of ≥2.5 mm, were included in the present study. A true CBL was described as stenosis of >50% in both the MB and the ostium of the SB according to the Medina classification (1,1,1; 1,0,1; and 0,1,1) [10] and could be located either in (1) the left anterior descending [LAD] artery and diagonal branch; (2) the left circumflex artery (LCx) and obtuse marginal branch (OM); or (3) the LMCA and LAD/LCx. Patients with the following criteria were excluded: (1) angiographic evidence of severely calcified LM lesions requiring atherectomy; (2) in-stent restenosis/thrombosis; (3) inherence to the procedural steps of optimization defined below as extracted from angiographic images and report reviews; (4) allergies to any of the drugs [aspirin, clopidogrel, ticagrelor, everolimus, zotarolimus, and sirolimus] used in the study; and (5) cardiogenic shock or unstable hemodynamics. Patients lost to follow-up were also excluded. Consequently, 142 patients were included in the final analysis. The flow-chart of the study is shown in Figure 1. The study protocol was approved by the institutional ethics committee (date: 26 September 2022; decision no: İ08-528-22). The requirement for informed consent was waived by the ethics committee due to the retrospective nature of the study. The study was conducted in accordance with the ethical principles outlined in the Declaration of Helsinki.

### 2.2. Procedures

Coronary procedures were performed using either a transfemoral and/or transradial approach. A 6 Fr arterial sheath was used for the transradial approach, and 6 Fr, 7 Fr, or 8 Fr arterial sheaths were used for the transfemoral approach. In some cases, we used a simultaneous dual vascular access site (radial–radial, radio-femoral, or femoro-femoral) that allowed simultaneous positioning of the two-stent delivery systems through two individual guiding catheters. All patients received loading doses of aspirin (300 mg) and clopidogrel (300 to 600 mg) or ticagrelor (180 mg) before or during the procedure. Intravenous unfractionated heparin 70–100 IU/kg was given at the start of the procedure. All patients were treated with DES. Different generations of DES were used, including zotarolimus eluting stents [Resolute, Medtronic], everolimus eluting stents including zotarolimus-eluting stents (Resolute, Medtronic, Minneapolis, MN, USA), everolimus-eluting stents (Xience, Abbott Vascular, Santa Clara, CA, USA), and biolimus-eluting stents (Biomatrix, Biosensors International, Singapore). Dual antiplatelet therapy was given for at least 12 months (aspirin: 100 mg once daily, clopidogrel: 75 mg once daily, or ticagrelor: 90 mg twice daily). Selection of treatment strategy, type of stenting technique, and use of glycoprotein (GP) IIb/IIIa receptor inhibitors were made at the discretion of the interventional cardiologist. Patients were divided into three groups according to different strategies, as follows: DK-crush, T/TAP, or mini culotte groups.

### 2.3. Bifurcation Stenting Techniques

The bifurcation stenting technique (DK-crush, mini-culotte, or T/TAP) was selected according to lesion anatomy and operator preference. All procedures were performed by experienced interventional cardiologists following the standard procedural steps and optimization principles described in the European Bifurcation Club consensus documents [9]. Final kissing balloon inflation (FKBI) and the proximal optimization technique (POT) were performed whenever appropriate according to the selected technique. Angiographic success was defined as the achievement of thrombolysis in myocardial infarction [TIMI] grade-3 flow, with a final residual diameter stenosis less than 20% in both MB and SB, and without flow limiting dissection. Procedural success was defined as angiographic success without the occurrence of a MACE during the hospital stay.

### 2.4. Follow Up

Clinical follow-up was performed by office visit or telephone at 30 days and every 3 months after discharge. Clinical events were recorded from the index PCI procedure until the end of the 6-month follow-up period. All patients underwent either telephone or hospital follow-up at a mean of 6 months. At 6 months, CCS grade and antianginal medication were also assessed. The primary end point of the study was a composite of cardiac death, myocardial infarction, stent thrombosis, and target vessel revascularization (TVR) at 6 months. Coronary angiography was performed if anginal symptoms developed and/or myocardial ischemia was detected on non-invasive imaging.

All major events were obtained by direct contact with the patient or their relatives, and rates of MACE were recorded. Clinical follow-up was carried out with office visits or telephone contacts. MACE was assessed during in-hospital stays and at a follow-up period of 6 months after the intervention. Stent thrombosis was classified according to the Academic Research Consortium’s definitions [11]. In the absence of angiographic evidence, both acute MI associated with the distribution of the coronary artery treated and any death not explained by other reasons were caused by stent thrombosis.

### 2.5. Statistical Analysis

Categorical variables are shown as frequencies and percentage values, and comparisons were made using chi-square or Fisher’s exact tests. Continuous variables are shown as means ± standard deviations (SDs), and these were compared between groups using an independent-t test or a Mann–Whitney U test, if appropriate. A comparison of means between multiple groups was performed using analysis of variance (one-way ANOVA) followed by Bonferroni’s post hoc tests. As only nine MACE events happened, conventional logistic regression was considered susceptible to model instability. Therefore, a Firth’s penalized-likelihood logistic regression was used to examine factors associated with 6-month MACE. Baseline serum creatinine and previous history of myocardial infarction were selected as clinically relevant baseline variables. Each variable was initially evaluated in a separate univariable Firth logistic regression model, after which both variables were entered simultaneously into a multivariable Firth logistic regression model. All probability values were two-sided, and the differences with probability levels (*p*) of less than 5% were considered significant. The statistical analyses were completed using the SPSS (version 21.0; IBM Corp, Armonk, NY, USA) software package.

## 3. Results

Overall, 38.7% of patients (n = 55) were treated by the DK-crush technique, 38.0% [n = 54] by the mini culotte technique, and 23.2% (n = 33) by the T/TAP technique. Patient demographics and clinical features according to stenting techniques are shown in Table 1. Most patients were male (87.3%, n = 124). More than half of the patients (54.2%, n = 77) had an elective presentation. There was a high prevalence of female gender and diabetes mellitus in the culotte group. Other demographic and laboratory features were similar among the groups. Medications and procedural characteristics are presented in Table 2.

**Figure 2:**
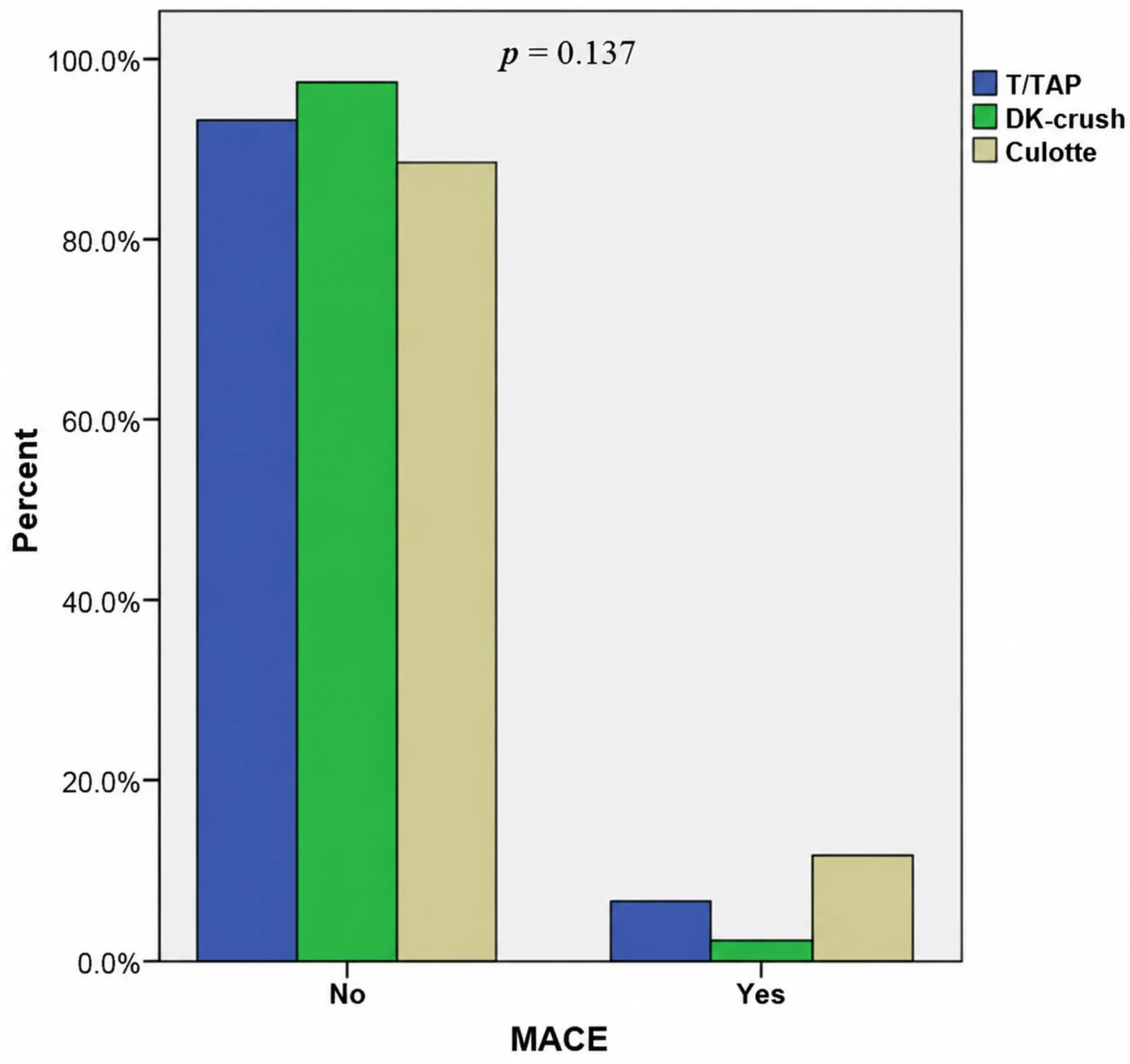
Comparison of different U2SSs in terms of 6-month MACE.

Prior and in-hospital drug therapies were similar among the groups. The bifurcation site was mostly in the LAD/diagonal region. As expected, bifurcation angle (>70°) was more frequent in the TAP group. Calcification ≥ moderate was more common in the DK-crush group (34.5%, n = 19). The mean stent diameter of the MV was smaller in the culotte group than in the other groups. Similarly, the amount of opaque used was significantly less in the culotte group. The cumulative MACE incidence was 6.3% (n = 9) (1 cardiac death, 4 TVR, 1 stent thrombosis, and 3 nonfatal MI) at a median follow-up period of 6 months. There was no significant difference in the primary clinical outcome among the groups. The DK-crush group had the lowest observed MACE rate; however, this difference was not statistically significant (*p* = 0.137) (Figure 2). Regardless of the selected technique allocated, freedom from MACE at 6 months was good, and all techniques were effective at relieving angina.

The clinical characteristics of patients in terms of MACE development are shown in Table 3 and Table 4. In the univariable Firth penalized logistic regression analyses, higher baseline serum creatinine was associated with an increased likelihood of MACE. Previous history of myocardial infarction was also associated with MACE (Table 5). However, in the multivariable Firth model, previous history of myocardial infarction remained the only variable associated with MACE (adjusted OR: 4.33; 95% CI: 1.10–23.61; *p* = 0.035).

## 4. Discussion

The current study included 142 patients with true CBL and involved a retrospective cohort created to evaluate the incidence and predictors of MACE at 6 months. The main findings of the present analysis are as follows: (1) the risk of the primary outcome was similar among the different U2SS in-patients who underwent coronary bifurcation stenting; (2) the DK-crush technique showed the lowest observed MACE rate among the evaluated strategies; however, the difference did not reach statistical significance; and (3) previous history of myocardial infarction may independently predict adverse outcome regardless of stenting technique after bifurcation U2SS.

CBLs account for approximately 15–20% of total PCI, of which true bifurcation lesions account for 30–40% [12]. The optimal PCI strategy for CBLs continues to be debated. For the interventional treatment of true CBLs, the procedure is complicated and difficult, and there are many intraoperative and postoperative complications that still represent a challenge for interventional cardiologists. Multiple randomized studies have assessed the clinical effects of provisional stenting versus U2SS for CBLs [13,14,15]. Most of these studies have shown that upfront U2SSs are associated with a higher risk of MACE than a provisional technique, which provides evidence-based data to update guidelines and expert recommendations, preferring provisional stenting for most CBLs. However, many cases of complex bifurcation lesions such as large caliber bifurcation lesions with significant ostial SB disease, or anticipated difficulty in accessing the SB after MV stenting, and distal true left main bifurcation cannot be treated by a one-stent strategy, and these complex lesions are best treated with a planned two-stent strategy [7,8,9]. Therefore, over the years, various double-stent bifurcation techniques have been developed, such as kissing-stent, stent-crush, culotte, T-stenting, Y-stenting, and double kissing crush [15,16,17,18,19]. However, in U2SSs, due to the complexity of the anatomical structures and the limitations of imaging, poor attachment and under expansion of the stent at the branch ostium, which cause in-stent thrombosis and restenosis, are common [20]. Besides, there is variability in how U2SSs are being performed, given the inconclusive data on the various steps of each strategy and long-term clinical outcomes [21,22,23,24,25].

Several trials have shown that DK-crush significantly reduces the risk of MACE for both non-left main-CBLs and left main-CBLs compared with provisional stenting, classic crush, or culotte [13,16,18]. However, intracoronary imaging and physiologic evaluation should be used to guide CBL interventions to achieve better clinical outcomes. Although we did not have the possibility of intracoronary imaging, the rate of MACE in our study was only 6.3%, a lower percentage than in previous studies. In the current study, the DK-crush group demonstrated the lowest observed MACE rate; however, no statistically significant difference was observed among the evaluated stenting strategies. Therefore, these findings should be interpreted with caution. Although procedural factors such as lesion complexity, routine use of final kissing balloon inflation (FKBI) and proximal optimization technique (POT), stent type, and contemporary dual antiplatelet therapy may have contributed to the observed numerical differences, the limited sample size and low number of clinical events preclude definitive conclusions regarding the comparative efficacy of the different techniques.

In the present study, no cases of acute or subacute stent thrombosis were observed, and only one patient experienced late stent thrombosis during the mean follow-up of 6 months. The cumulative MACE rate was 6.3% (n = 9). Although these event rates appear numerically lower than those reported in some previous studies, direct comparisons should be interpreted with caution because of differences in follow-up duration, patient characteristics, lesion complexity, procedural techniques, stent technology, and adjunctive pharmacological therapy [7,13,26].

Nevertheless, considering that stents in our cohort were deployed at a relatively higher mean inflation pressure (17.58 atm) than those reported in previous studies evaluating crush techniques, together with routine FKBI and systematic POT, these procedural optimization strategies may have contributed to the favorable outcomes. However, given the retrospective design, operator-dependent treatment allocation, and baseline heterogeneity, no causal relationship can be established, and these findings should be considered hypothesis-generating [26,27,28].

Taken together, in light of the current study, we have concluded that all upfront double stenting techniques are effective and safe as long as the stenting technique is chosen according to the operator’s experience and bifurcation lesion anatomy and is performed as recommended in the guidelines in accordance with the technique of each stage.

### Study Limitations

The present study has some shortcomings. Firstly, treatment allocation was based on operator discretion according to lesion anatomy and procedural complexity rather than randomization. Consequently, baseline lesion characteristics were not evenly distributed among the study groups, introducing the potential for selection bias and residual confounding. Therefore, comparisons between stenting strategies should be interpreted with caution. Furthermore, the relatively small sample size and low number of MACE events limited the statistical power to detect modest differences among the three strategies. Therefore, the absence of statistically significant differences should not be interpreted as evidence of equivalence. Rather, these findings should be considered hypothesis-generating and require confirmation in larger prospective multicenter studies. Although intravascular imaging is highly recommended for complex bifurcation PCI, both before stenting (to assess lesion composition and the need for pretreatment and to select the optimal stent sizes) and after stenting (to confirm adequate stent expansion, stent-strut apposition, and lack of stent distortion), intravascular imaging techniques were not used in the present study. Finally, because of the retrospective design, routine follow-up coronary angiography was not performed because it is not recommended in current clinical practice in the absence of recurrent symptoms or objective evidence of ischemia. Nevertheless, this approach may have resulted in underestimation of clinically silent restenosis or revascularization. In addition, the relatively short follow-up period limited the assessment of longer-term clinical outcomes, including late restenosis and very late stent thrombosis. Therefore, our findings should be interpreted as reflecting short-term clinical outcomes, and larger prospective studies with extended follow-up are warranted to validate these results.

## 5. Conclusions

Within the limitations of this retrospective single-center study, all upfront two-stent strategies appeared feasible and were associated with relatively low short-term MACE rates. However, larger prospective studies are required before definitive conclusions regarding comparative efficacy can be drawn.

## Figures and Tables

**Table 1:** Patient characteristics and laboratory measurements.

	T/TAP (n = 33)	DK Crush (n = 55)	DK Mini-Culotte (n = 54)	*p* Value
Age (years)	61.69 ± 9.97	61.09 ± 10.00	61.96 ± 10.52	0.902
Male gender	30 (90.9%)	51 (92.7%)	43 (79.6%)	0.094
Hyperlipidemia	24 (72.7%)	30 (54.5%)	34 (63%)	0.231
Hypertension	22 (66.7%)	41 (74.5%)	37 (68.5%)	0.682
Diabetes Mellitus	7 (21.2%)	19 (34.5%)	23 (42.6%)	0.126
Active Smoking	14 (42.4%)	28 (50.9%)	20 (37%)	0.577
Peripheral Artery Disease	4 (12.1%)	4 (7.3%)	3 (5.6%)	0.532
History of Cerebrovascular Disease	1 (3.0%)	1 (1.8%)	-	0.481
BMI kg/m^2^	28.41 ± 3.54	27.51 ± 3.58	28.25 ± 3.96	0.567
SBP, mm Hg	135.15 ± 15.59	133.93 ± 17.17	131.87 ± 15.93	0.636
DBP, mm Hg	81.82 ± 8.55	81.60 ± 7.99	80.24 ± 9.06	0.618
Heart rate (beat per min)	76.12 ± 14.67	76.07 ± 15.09	74.17 ± 9.67	0.705
Presentation at Admission (n, %)				
Stable Angina	13 (39.4%)	31 (56.4%)	33 (61.1%)	0.157
Non-STEMI	13 (39.4%)	18 (32.7%)	18 (33.3%)	
STEMI	7 (21.2%)	6 (10.9%)	3 (5.6%)	
LVEF (%)	47.58 ± 11.94	47.67 ± 9.63	51.61 ± 9.87	0.095
WBC Count (×10^9^)	9.87 ± 3.32	9.48 ± 3.68	9.19 ± 2.83	0.619
Platelet Count (×10^9^)	240.42 ± 44.82	256.69 ± 70.35	263.52 ± 72.84	0.289
Hemoglobin (g/dL)	13.98 ± 2.11	14.24 ± 1.48	14.03 ± 1.98	0.763
Serum Glucose (mg/dL)	123.30 ± 34.59	135.46 ± 54.60	133.68 ± 70.01	0.665
Creatinine (mg/dL) [median (IQR)]	0.92 (0.79–1.14)	0.9 (0.83–1.08)	0.93 (0.81–1.11)	0.449
eGFR (mL/min/1.73 m^2^)	77.55 ± 25.29	82.10 ± 20.47	80.35 ± 22.62	0.656
Total Cholesterol (mg/dL)	177.97 ± 48.53	178.19 ± 48.37	178.15 ± 51.12	1.000
LDL-C (mmol/L)	105.85 ± 46.13	104.98 ± 45.69	103.48 ± 44.86	0.970
Triglyceride (mmol/L)	170.45 ± 111.69	171.39 ± 111.80	158.59 ± 86.46	0.783
HDL-C (mmol/L)	41.06 ± 10.79	40.30 ± 8.21	42.02 ± 10.11	0.647
HbA1c (%)	6.65 ± 1.38	6.69 ± 1.46	7.01 ± 2.00	0.625
CRP (mg/L)	21.91 ± 41.38	13.22 ± 23.32	11.92 ± 17.89	0.268

BM, body mass index; SBP, systolic blood pressure; DBP, diastolic blood pressure; STEMI, ST-segment elevation myocardial infarction; LVEF, left ventricular ejection fraction; WBC, white blood cell; eGFR, estimated glomerular filtration rate; LDL-C, low-density lipoprotein cholesterol; HbA1c, Glycosylated hemoglobin; HDL-C, high-density lipoprotein; CRP, C-reactive protein; IQR, inter-quartile range.

**Table 2:** Medications and procedural characteristics.

	T/TAP (n = 33)	DK Crush (n = 55)	DK Mini-Culotte (n = 54)	*p* Value
Prior Aspirin Therapy	21 (63.6%)	39 (70.9%)	33 (61.1%)	0.543
Prior ACE-inhibitor Therapy	17 (51.5%)	29 (52.7%)	28 (51.9%)	0.993
Prior Beta-blocker Therapy	20 (60.6%)	33 (60%)	29 (53.7%)	0.746
Prior Statin Therapy	14 (42.4%)	32 (58.2%)	27 (50%)	0.347
In-hospital Aspirin Therapy	33 (100%)	55 (100%)	54 (100%)	-
In-hospital Beta-blocker Therapy				
Metoprolol	28 (84.8%)	45 (81.8%)	40 (74.1%)	0.422
Carvedilol	1 (3%)	2 (3.6%)	-	
Bisoprolol	3 (9.1%)	3 (5.5%)	8 (14.8%)	
Nebivolol	1 (3%)	2 (3.6%)	1 (1.9%)	
In-hospital ACE-inhibitor Therapy	30 (90.9%)	50 (90.9%)	48 (88.9%)	0.926
In-hospital P2Y12 Receptor Blockers Therapy				
Clopidogrel	11 (33.3%)	16 (29.1%)	13 (24.1%)	0.507
Ticagrelor	15 (45.5%)	23 (41.8%)	18 (33.3%)	
Prasugrel	7 (21.2%)	15 (27.3%)	21 (38.9%)	
In-hospital Statin Therapy	33 (100%)	54 (98.2%)	52 (96.3%)	0.498
Bifurcation Site				
LM/LAD/LCX	13 (39.4%)	10 (18.2%)	4 (7.4%)	0.001
LAD/Diagonal	14 (42.4%)	34 (61.8%)	27 (50%)	
LCX/OM	6 (18.2%)	11 (20%)	23 (42.6%)	
Medina Classification				
1.1.1	28 (84.8%)	43 (78.2%)	40 (74.1%)	0.532
1.0.1	4 (12.1%)	5 (9.1%)	8 (14.8%)	
0.1.1	1 (3%)	7 (12.7%)	6 (11.1%)	
Locations of Lesions				
Proximal	21 (63.6%)	44 (80%)	39 (72.2%)	0.297
Mid	12 (36.4%)	10 (18.2%)	13 (24.1%)	
Distal	-	1 (1.8%)	2 (3.7%)	
Multi-vessel Disease	33 (100%)	54 (98.2%)	54 (100%)	0.451
Bifurcation Angle (>70°)	33 (100%)	4 (7.3%)	1 (1.9%)	<0.001
Calcification ≥ Moderate	6 (18.2%)	19 (34.5%)	4 (7.4%)	0.002
Contrast-induced Nephropathy	2 (6.06%)	2 (3.64%)	5 (9.26%)	0.519
Mean Stent Diameter of MV (mm)	3.17 ± 0.43	3.02 ± 0.46	2.81 ± 0.40	0.001
Total Stent Length of MV (mm)	27.70 ± 6.16	27.51 ± 6.34	28.80 ± 8.10	0.602
Mean Stent Diameter of SB (mm)	2.67 ± 0.52	2.63 ± 0.40	2.69 ± 0.40	0.762
Total Stent Length of SB (mm)	20.64 ± 6.81	23.73 ± 8.49	22.87 ± 6.26	0.159
Number of Stents	2.10 ± 0.31	2.17 ± 0.47	2.19 ± 0.44	0.681
Opaque Amount (cc)	215.76 ± 41.46	216.91 ± 68.12	186.02 ± 54.49	0.011
Procedure Time (min)	120.00 ± 21.24	109.20 ± 24.06	109.20 ± 27.42	0.097
MACE	2 (6.1%)	1 (1.8%)	6 (11.1%)	0.137

ACE, angiotensin converting enzyme; LM, left main; LAD, left anterior descending; LCX, left circumflex; OM, obtus marginatus; MV, main vessel; SB, side branch; MACE, major adverse cardiac event.

**Table 3:** Clinical, procedural and laboratory characteristics of patients in terms of MACE.

	MACE (−) (n = 133)	MACE (+) (n = 9)	*p* Value
Age (years)	61.42 ± 10.094	63.88 ± 11.141	0.508
SBP, mmHg	133.51 ± 16.149	132.22 ± 19.221	0.819
DBP, mm Hg	81.14 ± 8.603	81.11 ± 7.407	0.993
Heart Rate (bpm)	75.53 ± 13.214	73 ± 12.196	0.578
LVEF (%)	49.03 ± 10.471	50 ± 10.00	0.788
White Blood Cell Count (×10^9^)	9.5709 ± 3.20990	7.8289 ± 1.36752	0.109
Hemoglobin (g/dL)	14.1399 ± 1.79873	13.5111 ± 2.25413	0.32
Platelet Count (×10^9^)	256.08 ± 67.709	247.11 ± 47.322	0.697
Serum Glucose (mg/dL)	132.22 ± 58.622	130.63 ± 47.032	0.94
Creatinine (mg/dL) [median (IQR)]	0.92 (0.81–1.085)	1.24 (1.04–1.405)	**0.002**
Total Cholesterol (mg/dL)	178.39 ± 49.490	174.22 ± 45.964	0.807
LDL-C (mg/dL)	104.656 ± 45.8423	103.889 ± 35.8066	0.961
Triglyceride (mg/dL)	165.45 ± 101.437	178.22 ± 119.213	0.718
HDL-C (mg/dL)	41.342 ± 9.4133	38.111 ± 11.7201	0.328
CRP (mg/L) [median (IQR)]	5.1 (2.0–13.0)	12.0 (6.45–17.5)	0.101
Mean Stent Diameter of MV (mm)	2.9756 ± 0.45108	3.0 ± 0.46771	0.876
Total Stent Length of MV (mm)	27.8120 ± 6.73655	31.4444 ± 10.06369	0.315
Mean Stent Diameter of SB (mm)	2.6523 ± 0.41610	2.7778 ± 0.56519	0.394
Total Stent Length of SB (mm)	22.9173 ± 7.38656	19.2222 ± 6.45712	0.146
Number of Stents	2.16 ± 0.411	2.2 ± 0.632	0.784
Inflation Pressure (atm)	17.62 ± 1.743	16.89 ± 1.054	0.214
BMI (kg/m^2^)	27.8646 ± 3.57009	29.2685 ± 5.83673	0.372
Procedure Time (min)	112.20 ± 25.02	106.80 ± 26.46	0.530

Statistically significant *p* values (*p* < 0.05) are shown in bold.

**Table 4:** Clinical characteristics and medications of patients in terms of MACE.

	MACE (−) (n = 133)	MACE (+) (n = 9)	*p* Value
Prior Myocardial Infarction	53 (39.8%)	7 (77.8%)	**0.036**
Peripheral Artery Disease	11 (8.3%)	-	0.473
Prior Cerebrovascular Disease	2 (1.5%)	-	0.877
Hyperlipidemia	65 (48.9%)	8 (88.9%)	**0.034**
In-hospital Beta-blocker Therapy			
Metoprolol	106 (79.7%)	7 (77.8%)	0.341
Carvedilol	2 (1.5%)	1 (11.1%)	
Bisoprolol	13 (9.8%)	1 (11.1%)	
Nebivolol	4 (3.0%)	-	
In-hospital ACE-inhibitor Therapy (n, %)	120 (90.2%)	8 (88.9%)	1.00
In-hospital P2Y12 Receptor Blockers Therapy			
Clopidogrel	37 (27.8%)	3 (33.3%)	0.648
Ticagrelor	54 (40.6%)	2 (22.2%)	
Prasugrel	39 (29.3%)	4 (44.4%)	
In-hospital Statin Therapy	131 (98.5%)	8 (88.9%)	0.18
Bifurcation Site (n, %)			
LM/LAD/CX	24 (18%)	3 (33.3%)	0.403
LAD/Diagonal	72 (54.1%)	3 (33.3%)	
LCX/OM	37 (27.8%)	3 (33.3%)	
Medina Classification			
1.1.1	103 (77.4%)	8 (88.9%)	0.579
1.0.1	16 (12%)	1 (11.1%)	
0.1.1	14 (10.5%)	-	
Locations of Lesions			
Proximal	95 (71.4%)	9 (100%)	0.173
Mid	35 (26.3%)	-	
Distal	3 (2.3%)	-	
Multi-vessel Disease (n, %)	132 (99.2%)	9 (100%)	0.937
Bifurcation Angle (>70°)	36 (27.1%)	2 (22.2%)	0.778
Presentation at Admission			
Stable Angina	72 (54.1%)	5 (55.6%)	0.506
Non-STEMI	45 (33.8%)	4 (44.4%)	
STEMI	16 (12.0%)	-	
Contrast-induced Nephropathy	5 (3.76%)	4 (44.44%)	**0.001**
Sex			
Male	117 (88%)	7 (77.8%)	0.319
Female	16 (12%)	2 (22.2%)	
Hypertension	93 (69.9%)	7 (77.8%)	0.471
Diabetes Mellitus	46 (34.6%)	3 (33.3%)	1.000
Smoking			
Yes	57 (42.9%)	5 (55.6%)	0.704
No	49 (36.8%)	3 (33.3%)	
Ex-smoker	27 (20.3%)	1 (11.1%)	
Calcification ≥ Moderate	29 (21.8%)	-	0.120

Statistically significant *p* values (*p* < 0.05) are shown in bold.

**Table 5:** Univariable and multivariable Firth analyses.

	Univariable Analysis			Multivariable Analysis		
	Odds Ratio	%95 CI	*p* Value	Adjusted Odds Ratio	%95 CI	*p* Value
Baseline Creatinine, per 1 mg/dL Increase	1.51	1.00–2.27	0.049 *	1.52	0.98–2.30	0.061
Previous MI	4.51	1.15–24.88	0.03 *	4.33	1.10–23.61	0.035 *

* Statistically significant (*p* < 0.05).

## Data Availability

The data presented in this study are available from the corresponding author upon reasonable request.
